# Effects of a Manual Response Requirement on Early and Late Correlates of Auditory Awareness

**DOI:** 10.3389/fpsyg.2019.02083

**Published:** 2019-09-10

**Authors:** Rasmus Eklund, Billy Gerdfeldter, Stefan Wiens

**Affiliations:** Gösta Ekmans Laboratorium, Department of Psychology, Stockholm University, Stockholm, Sweden

**Keywords:** auditory awareness negativity, late positivity, consciousness, response requirement, source analysis

## Abstract

In hearing, two neural correlates of awareness are the auditory awareness negativity (AAN) and the late positivity (LP). These correlates of auditory awareness are typically observed with tasks in which subjects are required to report their awareness with manual responses. Thus, the correlates may be confounded by this manual response requirement. We manipulated the response requirement in a tone detection task (*N* = 52). Tones were presented at each subject’s individual awareness threshold while high-density electroencephalography (EEG) activity was recorded. In one response condition, subjects pushed a button if they were aware of the tone and withheld responding if they were unaware of the tone. In the other condition, subjects pushed a button if they were unaware of the tone and withheld responding if they were aware of the tone. To capture AAN and LP, difference waves were computed between aware and unaware trials, separately for trials in which responses were required and trials in which responses were not required. Results suggest that AAN and LP are unaffected by the response requirement. These findings imply that in hearing, early and late correlates of awareness are not confounded by a manual response requirement. Furthermore, the results suggest that AAN originates from bilateral auditory cortices, supporting the view that AAN is a neural correlate of localized recurrent processing in early sensory areas.

## Introduction

How does the brain enable the experience of seeing a picture or hearing a tone? This question has been investigated with threshold tasks (Koivisto and Revonsuo, 2010). In these tasks, a stimulus is presented at the individual awareness threshold: the level at which a subject reports being aware of the stimulus half of the time. If neural activity is recorded during the task, the contrastive analysis of the difference in neural activity between trials rated as aware and trials rated as unaware reflects the neural correlate of consciousness (NCC; Aru et al., 2012).

NCCs have been studied with event-related potentials (ERPs). These potentials are time-locked responses derived from electroencephalography (EEG). As such, they have excellent temporal resolution (Luck, 2014). In vision, two ERPs have been reported (Koivisto and Revonsuo, 2010; Eklund and Wiens, 2018): a negative difference wave about 200 ms after visual onset at occipital electrodes [visual awareness negativity (VAN)], and a positive difference wave about 300 ms after visual onset at parietal electrodes [late positivity (LP)].

Importantly, we recently found that in threshold tasks ERP correlates of awareness in hearing resemble those in vision (Eklund and Wiens, 2019). Specifically, we found a negative difference wave at about 200 ms after auditory onset at frontocentral electrodes (auditory awareness negativity, AAN), and a positive difference wave about 300 ms after auditory onset at parietal electrodes (LP). These findings from vision and hearing provide evidence for similar correlates of awareness: an early correlate (VAN in vision and AAN in hearing) and a late correlate (LP).

However, these NCCs may be confounded by a response requirement (Tsuchiya et al., 2015). For example, because in typical thresholds tasks, subjects are required to report awareness with button presses for every trial, the NCCs may be confounded by this requirement for a manual response. If so, neural activity to trials rated as aware minus neural activity to trials rated as unaware might also reflect activity other than awareness.

In vision, Koivisto et al. (2016) devised a task to separate the neural activity of responding manually from that of awareness. On each trial, a Gabor patch (Gaussian filtered grating) was presented at the individual awareness threshold, and subjects reported their awareness of the Gabor in two different response conditions. In one condition, subjects pushed a button if they were aware of the Gabor and withheld responding if they were unaware of the Gabor. The other condition was reversed: subjects pushed a button if they were unaware of the Gabor and withheld responding if they were aware of the Gabor. From these conditions, two difference ERPs were calculated: the *response ERP* and the *no-response ERP*. The response ERP was computed from trials in which subjects pressed a button to indicate either awareness or unawareness. Thus, the response ERP captured the difference of aware minus unaware trials for response trials. The no-response ERP was computed from trials in which subjects did not press a button to indicate either awareness or unawareness. Thus, the no-response ERP captured the difference of aware minus unaware trials for no-response trials.

Koivisto et al. (2016) argued that if the requirement to press a button confounds the NCCs, then there should be a difference between the response ERP and the no-response ERP. In their study, there was no statistically significant difference between VAN to response trials and VAN to no-response trials. According to the authors, this lack of a statistically significant difference suggests that VAN was unaffected by the requirement to press a button. Results also showed that the LP (recorded between 350 and 450 ms) was significantly larger to response trials than no-response trials. Because awareness should precede the preparation of a manual response to report this awareness, the finding that LP was affected by the response requirement suggests that LP is not a pure measure of awareness. According to the authors, these findings suggest that VAN reflects visual awareness whereas LP reflects post-perceptual processes.

In a related study in vision, subjects detected a Gabor patch at the awareness threshold as neural activity was measured with ERP (Ye and Lyu, 2019). Subjects responded manually either immediately after each visual stimulus or after a 2-s delay. The analyses showed that the response manipulation had no statistically significant effect on either VAN or the LP at 450–650 ms. However, an exploratory analysis suggested a statistically significant effect on the LP at 650–850 ms (note that this analysis was exploratory because it was conducted only after the first round of review). At this later interval, LP amplitude to delayed responses was more positive than LP amplitude to immediate responses. Presumably, LP was sustained because of delayed responding. Because the LP between 650 and 850 ms was affected by the response task and the VAN was not, the authors concluded—similar to Koivisto et al. (2016)—that VAN reflects visual awareness, whereas LP reflects post-perceptual processing.

In hearing, it is unresolved whether manual responding confounds the neural correlates of awareness. Two early studies recorded EEG in an active detection task and in a passive task (Hillyard et al., 1971; Squires et al., 1973). In the active detection task, subjects pressed different buttons to indicate whether they detected a tone. Results suggested an early negativity and a late positivity to detected compared to undetected tones. Thus, early and late neural correlates of detection were obtained when subjects had a manual response requirement. In the passive task, tones of varying intensity were played while subjects either sat passively (Hillyard et al., 1971) or read a book (Squires et al., 1973). The awareness of tones was not assessed. Nonetheless, if subjects detected many of the tones and these detected tones elicited an early negativity and a late positivity, then an overall early negativity and an overall late positivity should be obtained across all tones (i.e., for the averages across detected and undetected tones). However, results across tones did not suggest an early negativity and late positivity. These findings suggest that the neural correlates of detection may have been eliminated when subjects did not respond manually to the tones. However, these differences may have been obtained because of the requirement to listen for the tones in the active task but not in the passive task, rather than because of differences in the manual response requirement.

In support of this supposition, a more recent study (Shucard et al., 2004) suggests that listening for target syllables and a manual response requirement have independent effects on the P3, which overlaps in time with the LP. Subjects were presented with a stream of various syllables and performed two tasks: They either pushed a button if they detected a target syllable or simply listened for a target syllable. Although P3 amplitudes were largest to targets that required a button press, P3 amplitudes were also larger to targets than non-targets when subjects simply listened for a target syllable. This suggests that listening for tones increases P3 amplitudes to the tones and that an additional response requirement increases P3 amplitudes even further. If so, the neural correlates of detection may have been eliminated in previous studies (Hillyard et al., 1971; Squires et al., 1973) because tones were not listened for, and not because of the lack of a manual response requirement.

With regard to the early neural correlate of auditory awareness, results of a multitone masking task in a magnetoencephalography (MEG) study suggest that an awareness-related negativity can be obtained without a manual response requirement (Gutschalk et al., 2008). Subjects were instructed to detect a target tone that was repeated within masking background tones and to press a button as soon as they detected the target. A negativity was observed about 200 ms after target-tone onset (awareness-related negativity), but only after subjects had detected the targets (and were presumably aware of them). A passive task was also included in which subjects were sometimes cued with unmasked target tones before a trial (to facilitate the subjects’ awareness of the targets). Results showed a larger negativity to cued tones than uncued tones, suggesting that the awareness-related negativity can be obtained even if a manual response is not required. Notably, the study did not measure the P3 and did not perform a contrastive analysis.

Because previous evidence is limited, the main goal of the present study was to examine the effects of a manual response requirement on AAN and LP in an active listening task. To increase the evidential strength of our data, we preregistered the hypotheses, method, and analyses^1^. Furthermore, previous studies interpreted a nonsignificant effect of response requirements on the VAN (Koivisto et al., 2016; Ye and Lyu, 2019) and the LP between 450 and 650 ms (Ye and Lyu, 2019) as evidence for no difference between response conditions. Critically, a nonsignificant result does not necessarily prove the null hypothesis of no difference between the two conditions (Amrhein et al., 2019). For example, if the studies did not have enough power because of small sample sizes, then a nonsignificant result is not surprising but expected. Because *a priori* power analyses were not conducted, the nonsignificant results are not diagnostic. In contrast, Bayesian hypothesis testing can be used to compare two models (null hypothesis and alternative hypothesis) and thus provide evidence for or against the null hypothesis (Dienes, 2008, 2016; Wiens and Nilsson, 2017; Wagenmakers et al., 2017b). Therefore, we conducted Bayesian analyses to measure the strength of evidence for or against the null hypothesis.

Another goal of our study was to explore the neural generators of AAN. In our previous study (Eklund and Wiens, 2019), no source localization could be attempted because too few electrodes were recorded. In the MEG study of the multitone masking task (Gutschalk et al., 2008), the source of the awareness-related negativity appeared to be bilateral auditory cortices. This negativity was located in the auditory cortex and occurred 200 ms after stimulus onset. Because it is plausible that AAN has the same neural generator, we used a high-density electrode array with a sufficient number of electrodes to conduct source localization.

In summary, we used a tone detection task and manipulated the response requirement of reporting awareness in two conditions. Tones were presented at each subject’s individual awareness threshold, and neural activity related to awareness was measured. Trials with manual responses were compared with trials in which no manual responses were required. We also recorded high-density electroencephalography to explore the neural generators of AAN.

## Method

The method and analyses were preregistered before any data were collected (see text footnote 1). Deviations from the preregistration are noted below. All data, scripts, and supplementary files are available at a university depository (Wiens and Eklund, 2019).

### Participants

We preregistered to recruit at least 20 subjects. If the Bayes Factor (BF) exceeded 3 or was below 1/3 for our hypotheses, recruitment would end. Otherwise, recruitment would continue until the BF reached the criterion, until a maximum of 50 subjects were retained after exclusion, or at the end of June 2019.

The final sample consisted of 52 healthy subjects (age: *M* = 27.8 years, SD = 4.9), of whom 18 were male and 46 were right-handed. Subjects were recruited from local universities and through online billboards. Recruitment stipulated a target age range of 18–40 years, no history of neurological diseases, normal or corrected to normal vision, and normal hearing. Participation was compensated with gift vouchers. Ethical review and approval was not required for the study on human participants in accordance with the local legislation and institutional requirements. Participants provided their written informed consent to participate in this study.

One subject was excluded because of excessive noise in half of the electrodes. Although we did not preregister any behavioral exclusion criteria, two subjects were excluded for the following reasons: one subject did not respond as instructed (i.e., pressed up and down arrows instead of spacebar), and another subject stopped the task half way through the experiment.

### Apparatus and Stimuli

The stimulus was a 100-ms tone (*f* = 1,000 Hz, 5 ms fade-in and fade-out). The tone was presented through in-ear tubephones (ER2; Etymotic Research Inc., IL; www.etymotic.com). Instructions were displayed on a BenQ XL2430T, 24-inch gaming monitor (at 144 Hz, 1,920 × 1,080 resolution). PsychoPy v 3.0 (Peirce, 2007) was used to generate the tone and to collect behavioral data. A Cedrus StimTracker (Cedrus Corporation, San Pedro, CA) was used to track tone onsets. This compensated for any timing errors between the event marker from the presentation computer and the actual presentation of a tone.

### Procedure

**Figure 1:**
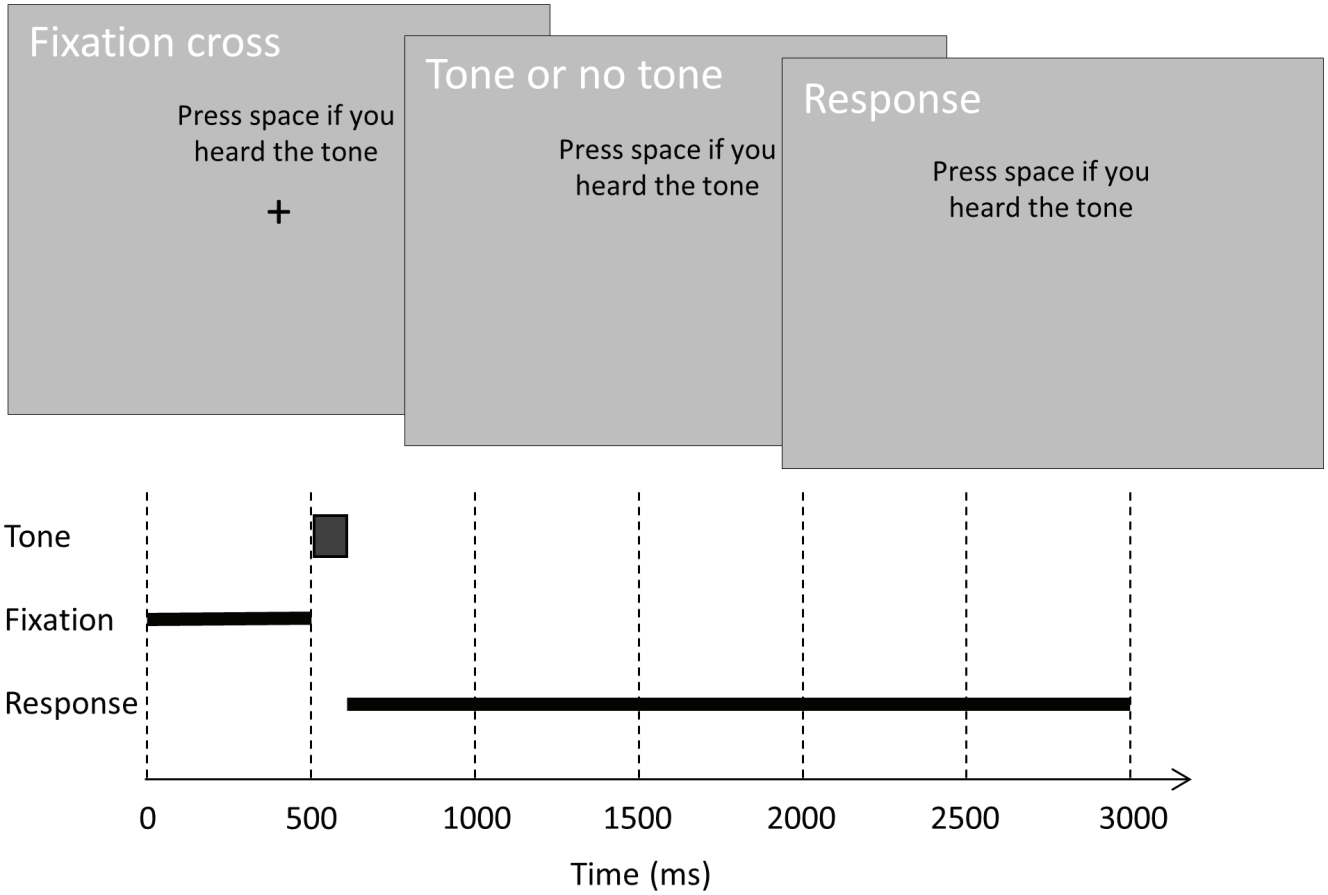
The time course of a trial. On each trial, a black fixation cross (0.5°) was displayed on a gray background for 500 ms. On critical and control trials, a tone was played binaurally at fixation offset. On catch trials, no tone was played. Subjects had 2,400 ms to rate their subjective awareness of the tone. In one condition, subjects were instructed to press the spacebar if they heard the tone. In the other condition, subjects were instructed to press the spacebar if they did not hear the tone.

Subjects performed a tone-detection task while seated in front of a computer screen with their chin in a chinrest. Figure 1 shows the time course of a trial. On each trial, a 500-ms black fixation cross (0.5 visual degrees) was followed by a 100-ms tone and a 2,400-ms interval to allow for a speeded response. Throughout the trial, the response instruction was displayed above the fixation cross to remind subjects of the current response requirement. Note that in the preregistration, we incorrectly stated that subjects had 3,000 ms rather than 2,400 ms to respond after tone offset. We also failed to preregister that the instruction was displayed continuously above the fixation cross.

Critical trials contained a tone at the individual subject’s auditory awareness threshold, and control trials contained a tone 10 dB above the individual awareness threshold. On these trials, the tone was played binaurally 500 ms after trial onset (i.e., at fixation cross offset). On catch trials, no tone was played. Before each block, subjects received one of two instructions on how to respond. In one condition, they were instructed to press the spacebar if they heard a soft tone (and not to press the spacebar if they did not hear a soft tone). In the other condition, they were instructed to press the spacebar if they did not hear a soft tone (and not to press the spacebar if they heard a soft tone). These instructions alternated over blocks, and the starting instruction alternated over consecutive subjects.

The task comprised 600 trials (480 critical, 60 control, and 60 catch). The trials were divided into six blocks of 100 trials (80 critical, 10 control, and 10 catch). The order of the trials was randomized within each set of 10 trials (8 critical, 1 control, and 1 catch). A self-paced break was allowed between blocks.

Before the experiment, subjects performed a short practice task. A fixation cross was displayed for 500 ms, followed by a clearly audible tone. Afterward, subjects rated their subjective awareness of the tone by using two buttons (the up arrow corresponding to “I heard the tone weakly”, and the down arrow corresponding to “I did not hear the tone”). After the practice task, interleaved staircases were used to calibrate the tone to an intensity that the subject reported hearing on approximately 50% of the trials (individual auditory awareness threshold). The staircase procedure consisted of three interleaved staircases. One staircase started at 4 dB. The other two staircases started at 20 dB above and below the first. The staircase procedure was as follows: If the subject reported hearing a tone, the level decreased. If the subject reported not hearing a tone, the level increased. For each staircase, reversal steps were 8, 8, 4, 4, 2, and 2 dB for the first six reversals and were 1 dB for the subsequent reversals. Every separate staircase stopped after 12 reversals. Within a set of three trials, one trial was presented from each staircase in random order. If a staircase was completed before the others, only the remaining staircases were sampled.

After the calibration, a validation block was run with 50 tone trials. The level of the tone in the validation block was determined from a psychometric response function generated from an individual subject’s staircase data. If the subject did not rate close to 50% of tones as aware, the tone level was adjusted according to the psychometric response function that estimated the individual awareness threshold on the basis of all data gathered so far. If necessary, validations were repeated to allow the threshold estimate for the tone level to stabilize at 50%. The preregistration stated that if the 50% threshold was not reached after four blocks of validation, the subject would be tested at a tone level that seemed most promising in capturing the individual auditory awareness threshold. However, eight subjects were tested with more than four blocks (max = 6) of validation before deciding about the tone level. In the final sample, the mean number of validations was *M* = 3.15 (SD = 1.13).

### Electroencephalography Recording

EEG data were recorded from 64 electrodes at standard 10–20 positions, one electrode on the tip of the nose, and one on the right cheek with an Active Two BioSemi system (BioSemi, Amsterdam, Netherlands). An EEG cap (Electro-Cap International, Eaton, OH) was used to position the 64 electrodes together with two additional, system-specific electrodes. CMS (common mode sense, between PO3 and POz) served as the internal reference electrode, and DRL (driven right leg, between POz and PO4) served as the ground electrode. These 64 positions were recorded with pin electrodes, and the tip of the nose and cheek were recorded with flat electrodes attached with adhesive disks. Data were sampled at 1,024 Hz and filtered with a hardware low-pass filter at 104 Hz.

### Data Analysis

The data were processed and analyzed with MNE Python (Gramfort et al., 2013, 2014) and R (R Core Team, 2016). EEG data were processed offline. The behavioral analyses included all trials, whereas in the EEG data analyses, some trials were excluded (see below). In the EEG data analyses, tone onset was indexed by a Cedrus StimTracker to eliminate any timing errors in tone onset. EEG data were preprocessed as described in the preregistration. Offline, continuous EEG data were high-pass filtered with a 1-Hz Butterworth 4th degree two-pass filter. All electrodes were re-referenced to the tip of the nose, and Fpz was also re-referenced to the cheek electrode (for a combined measure of vertical and horizontal electrooculography). Individual EEG electrodes were visually inspected to detect noisy electrodes. Any noisy electrodes were interpolated (spherical spline interpolation) from neighboring electrodes (*M* = 0.44, SD = 0.83). Eye-blinks were corrected with ICA (fastica). Before ICA, the continuous EEG data were preprocessed as follows: Pauses were removed, noisy channels were interpolated, and a 1-Hz high-pass filter was applied. ICA (fastica) was conducted and eyeblink components were selected by manual inspection of their topography. The number of components removed per subject were *M* = 1.08 (SD = 0.33). For all trials, epochs were extracted from 100 ms before tone onset to 600 ms after tone onset. Each epoch was baseline corrected to the mean of the 100-ms interval before tone onset (−100 to 0 ms). For each subject, maximum amplitude ranges were extracted for individual epochs, and the distribution of these amplitude ranges was inspected. Individual trials that were apparent outliers were excluded. The number of trials removed per subject were *M* = 25.92 (SD = 20.00), corresponding to *M* = 4.32 (SD = 3.33) percent. The exclusion thresholds were set for each individual because subjects showed substantial variability in these amplitude ranges. Critically, inspection of trials was blinded to trial type (critical, control, catch, or response) and awareness ratings to avoid bias (Keil et al., 2014).

### Event-Related Potential Analysis

Four event-related potentials (ERPs) were derived from critical trials on the basis of the response condition and the awareness rating given by each subject. *Aware response* trials were tones rated as aware by pressing the spacebar. *Unaware response* trials were tones rated as unaware by pressing the spacebar. *Aware no-response* trials were tones rated as aware by not pressing the spacebar. *Unaware no-response* trials were tones rated as unaware by not pressing the spacebar. For each response condition, a difference wave was calculated by subtracting the unaware ERP from the aware ERP, resulting in two difference ERPs: the response ERP and the no-response ERP. For both ERPs, we preregistered that there would be a negativity between 160 and 260 ms after tone onset (AAN) and a positivity (in the P3 interval) between 350 and 550 ms after tone onset (LP). The AAN and LP intervals match those used in our previous study (Eklund and Wiens, 2019). For analysis of AAN and LP, electrodes were selected on the basis of preliminary results from an unpublished study (Eklund et al., 2019, unpublished). In this study, subjects discriminated between tones (low and high pitch) and rated their awareness. When only correct responses were considered, the difference ERP between aware and unaware trials (across pitch) suggested that the AAN and the LP had their peaks at a similar central-parietal location, as discussed in a supplementary file (Wiens and Eklund, 2019). Because we expected a similar topography in the present study, mean AAN and LP amplitudes were computed across a set of 15 central-parietal electrodes (C3, C1, Cz, C2, C4, CP3, CP1, CPz, CP2, CP4, P3, P1, Pz, P2, and P4).

We conducted Bayesian hypothesis testing to determine the degree of evidence for or against the alternative hypothesis (Dienes, 2008). The Bayes Factor (BF_10_) expresses the likelihood of the data given the alternative hypothesis relative to the likelihood of the data given the null hypothesis, whereas the BF_01_ shows the reverse (Dienes, 2008, 2016; Wiens and Nilsson, 2017; Wagenmakers et al., 2017b). Although the BF is a continuous measure of evidence, we adopted a common interpretation (Wagenmakers et al., 2017a). According to this interpretation, 1 < BF < 3 is anecdotal (or inconclusive) evidence, 3 < BF < 10 is moderate evidence, 10 < BF < 30 is strong evidence, 30 < BF < 100 is very strong evidence, and BF > 100 is extreme evidence. The BF was calculated with scripts (Wiens, 2017) that compute and plot the BF for mean differences in raw units if the alternative hypothesis is modeled as a normal, *t*, or uniform distribution, and the likelihood is modeled as a normal or *t* distribution (Dienes and McLatchie, 2018). For all hypotheses, one-sample Bayesian *t* tests were computed with the alternative hypothesis (or prior) modeled as a uniform distribution with the limits between −1 and +1 μV. We used BF greater than 3 or less than 1/3 as the cutoff. Furthermore, we computed the 95% confidence intervals (with an uninformed prior) for the mean amplitudes.

### Source Localization

**Figure 2:**
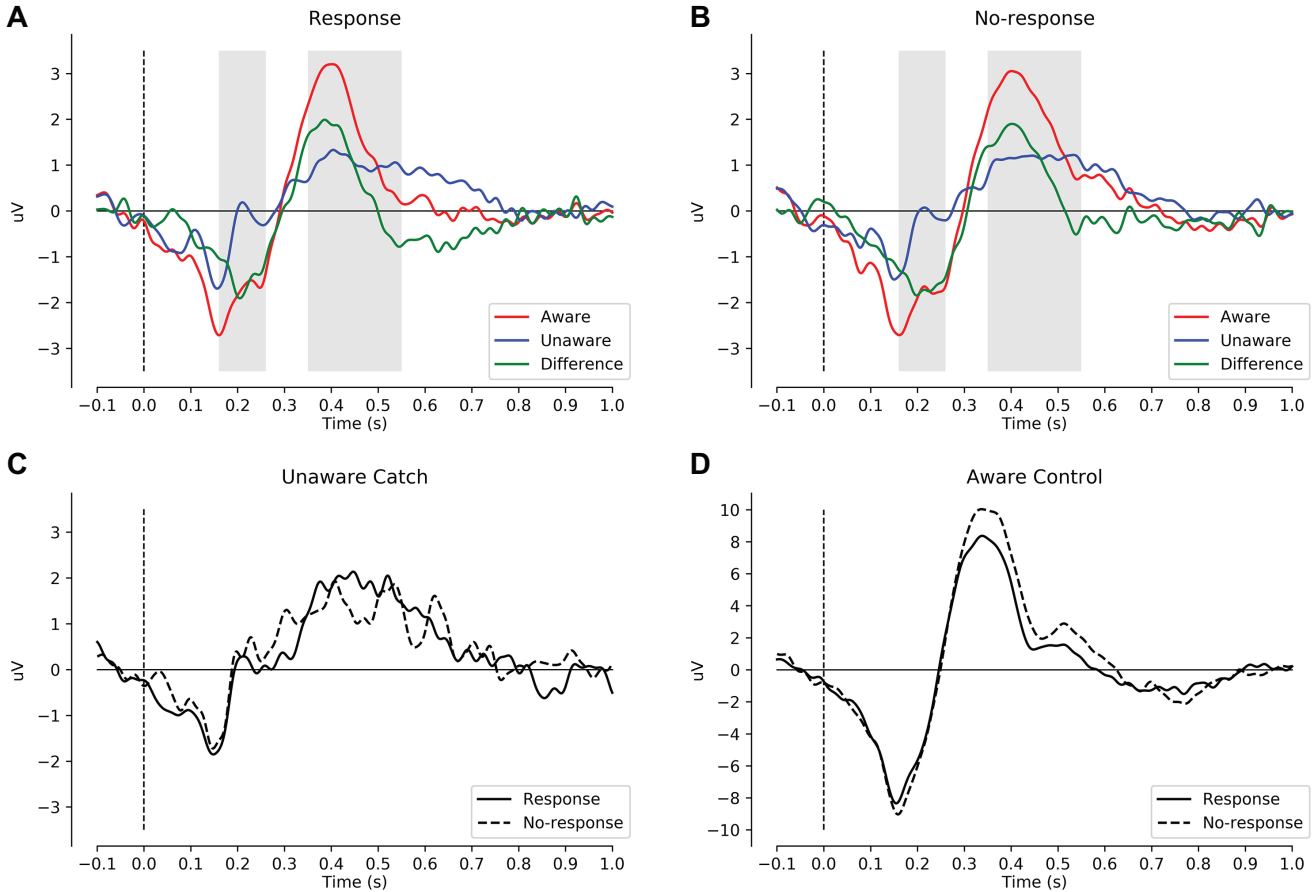
Mean ERPs across the preregistered 15 central parietal electrodes. **(A)** ERPs to critical tones for response trials: aware (red), unaware (blue), and aware minus unaware (green). The gray intervals mark the preregistered intervals for AAN (160–260 ms) and LP (350–550 ms). **(B)** ERPs to critical tones for no-response trials. **(C)** Catch trials that subjects rated as unaware by responding (solid) or not responding (dashed). **(D)** Control tones that subjects rated as aware by responding (solid) or not responding (dashed). In these plots, the data were low-pass filtered at 30 Hz. AAN, auditory awareness negativity; LP, late positivity.

To explore the generators of the AAN scalp topography, we performed source analysis with dynamic statistical parametric mapping (Dale et al., 2000) as implemented in MNE-Python (Gramfort et al., 2013, 2014; Andersen, 2018). Because individual magnetic resonance images were not available, a template brain from MNE-Python was used to model both the cortex (sources 3.1 mm apart) and the volume conductor (a boundary element method that models brain, skull, and skin separately with unique conductivities). To capture the AAN, source localization was performed on the mean ERP difference between aware and unaware critical trials across response and no-response trials at 210 ms after tone onset, which was the peak of AAN (see green lines in Figures 2A,B). Further, because clearly audible tones (i.e., control tones that subjects rated as aware) should elicit auditory cortex activation, source localization was also performed on the mean ERP across control tones at 150 ms, which was the peak of the auditory N1 (see Figure 2D). For completeness, we also performed source localization on the peak of the LP at 400 ms (similar to AAN) and the peak of the P3 at 320 ms (similar to N1). Because source localization was explorative, no significance testing was performed.

## Results

### Behavior

Table 1 shows the descriptive statistics for the behavioral data. Subjects performed the task as intended: Most control tones were rated as aware, and most catch trials were rated as unaware. Critically, close to 50% of the critical tones were rated as aware. Thus, tones were presented at the individual awareness threshold (50%). For the critical tones, mean reaction time to tones rated as unaware was 754 ms (SD = 152) and to tones rated as aware 681 ms (SD = 133); thus, subjects responded more slowly to tones rated as unaware than aware, mean difference = 73 ms, 95% CI [33, 113]. Across participants, the mean sound pressure level of the critical tone in the experiment was 6 dB (SD = 4).

**Table 1:** Descriptive statistics (Mean and SD) of awareness ratings.

	Response trials		No-response trials		Difference	
	Mean	SD	Mean	SD	Mean	95% CI
Critical: aware (%)	43.1	16.4	46.4	17.2	−3.2	[−6.5, 0.1]
Critical: unaware (%)	53.6	17.2	56.9	16.4	−3.2	[−6.5, 0.1]
Control: aware (%)	98.4	3.7	96.7	5.0	1.7	[0.3, 3.0]
Catch: aware (%)	6.4	6.5	9.0	8.7	−2.6	[−5.1, −0.1]

*The behavioral data show that subjects performed the task as instructed for response trials and no-response trials: Subjects indicated being aware of close to 50% of the tones at the awareness threshold (critical trials). They rated to be aware of most of the tones above the threshold (control trials) and rated to be aware on only a few trials when there was no tone (catch trials)*.

### Auditory Awareness Negativity: Event-Related Potential

**Figure 3:**
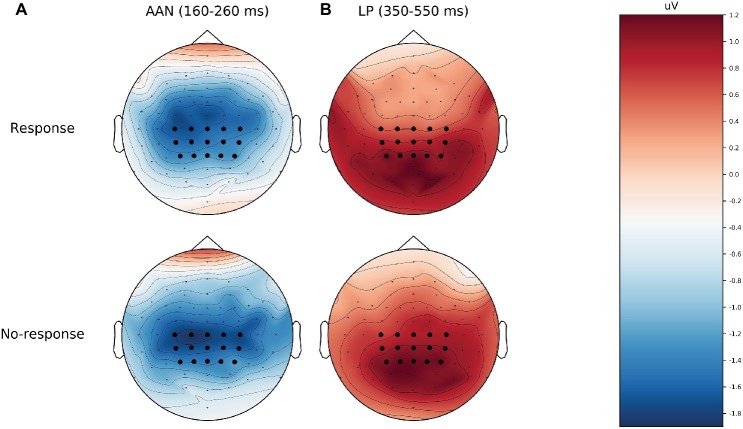
Topographies of the mean amplitude difference between aware and unaware for response (top) and no-response trials (bottom). **(A)** AAN, from 160 to 260 ms after tone onset. **(B)** LP, from 350 to 550 ms after tone onset. The preregistered electrodes are marked as black dots. AAN, auditory awareness negativity; LP, late positivity.

Table 2 shows the descriptive and inferential statistics for the mean amplitudes for AAN and LP; for the number of trials per condition, see supplementary data files (Wiens and Eklund, 2019). Figure 2 shows mean ERPs across all subjects. In Figures 2A,B, the difference between aware and unaware trials (green line) shows a negativity with a peak at 200 ms after stimulus onset for response trials Figure 2A and for no-response trials Figure 2B, suggesting AAN for each response requirement. Figure 3 shows topographies of the aware trials minus unaware trials, separately for response trials (first row) and no-response trials (second row). Figure 3A shows that AAN has a negativity at central electrodes for both response and no-response trials between 160 and 260 ms after tone onset. For the preregistered prior, Bayesian *t* tests confirmed the presence of AAN to response trials (BF_10_ > 20,000) and to no-response trials (BF_10_ > 7,000). However, there was inconclusive evidence for or against a difference in mean AAN amplitude between response trials and no-response trials (BF_01_ = 2.0). Because our preregistered prior (of a difference between −1 and +1 μV between conditions) was reasonable but arbitrary, we also explored another prior: We applied the default prior (Cauchy = 0.707) that is recommended for standardized effects and used in the software JASP (Wagenmakers et al., 2017a,b). Notably, the evidential strength was moderate in support of no difference between response and no-response trials (BF_01_ = 5.9). Furthermore, as shown in Figure 3, AAN was more anterior than the preregistered electrodes. Therefore, we repeated the above analyses with a data-driven electrode selection (Wiens and Eklund, 2019). Results were similar to those with the preregistered electrode selection. For example, with the default prior, the evidence suggested no difference between response and no-response trials for AAN (BF_01_ = 6.3).

**Table 2:** Descriptive and inferential statistics for the mean amplitude differences of aware trials minus unaware trials.

ERP	Condition	*N*	Mean (μV)	SD	95% CI	BF_01_	BF_10_
AAN (160–260 ms)							
	Response	52	−1.45	1.65	[−1.91, −0.99]	0.01	28109.21
	No-response	52	−1.62	1.88	[−2.14, −1.09]	0.01	7757.17
	Difference	52	0.17	2.52	[−0.53, 0.87]	2.04	0.49
LP (350–550 ms)							
	Response	52	0.83	1.40	[0.45, 1.22]	0.01	628.23
	No-response	52	1.00	1.22	[0.66, 1.34]	0.01	74926.39
	Difference	52	−0.16	1.87	[−0.68, 0.36]	2.50	0.40
LP (350–450 ms)							
	Response	52	1.72	1.73	[1.23, 2.20]	0.01	45004.38
	No-response	52	1.65	1.55	[1.22, 2.08]	0.01	268716.90
	Difference	52	0.06	2.23	[−0.56, 0.68]	2.50	0.40

*The 95% CI is the 95% confidence interval with a flat prior. BF_01_ and BF_10_ refer to the Bayes factor with the preregistered prior of a difference between −1 and +1 μV. AAN, auditory awareness negativity; LP, late positivity*.

To simulate the effect of even larger sample sizes on the Bayes Factor (with the preregistered prior), we assumed that our sample was representative of the population and sampled randomly (with replacement) from this population. For various sample sizes (between 50 and 300), we ran 10,000 simulations each, used the preregistered prior and electrodes, and computed the mean BF_01_ and its 95% CI across the simulations for each sample size (Wiens and Eklund, 2019). Results suggested that even 300 subjects might not be enough to yield moderate support for no difference between response and no-response trials (BF_01_ = 2.7).

Close inspection of Figures 2A,B suggested a very early negativity (at 160 ms after tone onset) to critical tones irrespective of awareness and the response requirement. This negativity had a bilateral occipital topography (Wiens and Eklund, 2019). Because this negativity was apparent also in catch trials in which no tones were presented (see Figure 2C), it was probably a visual response generated by the offset of the fixation cross, which occurred simultaneously with tone onset. Figure 2D shows the ERPs to control tones that subjects rated as aware. For these trials, a large N1 and P3 were obtained (note the different scaling for control tones in Figure 2).

### Auditory Awareness Negativity: Source Localization

**Figure 4:**
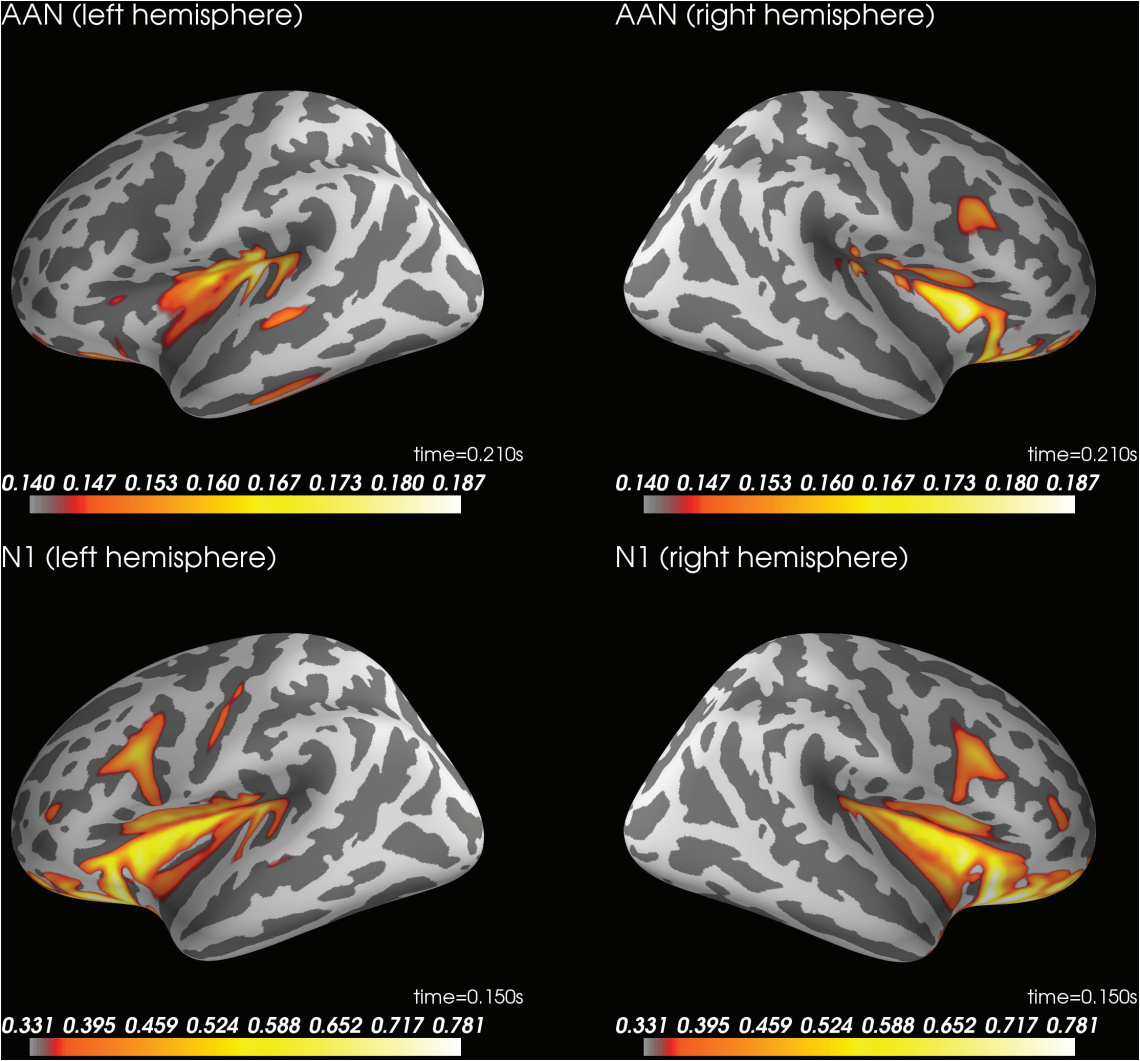
Source localization for AAN at 210 ms after stimulus onset (top row) and N1 to control trials rated as aware at 150 ms after stimulus onset (bottom row). AAN, auditory awareness negativity.

Figure 4 shows the results of the source localization. For AAN, source localization suggested activity in bilateral auditory cortices (superior temporal cortex). For the N1 to control tones, source localization suggested similar locations. See supplementary files for videos of the time course (Wiens and Eklund, 2019).

### Late Positivity: Event-Related Potential

In Figures 2A,B, the difference between aware and unaware trials (green line) shows a positivity with a peak at 400 ms after stimulus onset for response trials Figure 2A and for no-response trials Figure 2B, suggesting LP for each response requirement. Figure 3B shows that LP has a positivity at parietal electrodes for response trials and for no-response trials between 350 and 550 ms after tone onset. For the preregistered prior, Bayesian one-sample *t* tests confirmed the presence of LP for response trials (BF_10_ > 600) and no-response trials (BF_10_ > 70,000), see Table 2. However, there was inconclusive evidence for or against a difference in mean LP amplitude between response trials and no-response trials (BF_01_ = 2.5). In contrast, when we explored the BF for a default prior (Cauchy = 0.707), the evidential strength was moderate in support of no difference between response and no-response trials (BF_01_ > 5.5).

Visual inspection of Figure 2 suggested that the actual LP was shorter (between 350 and 450 ms) than assumed in the preregistered interval (between 350 and 550 ms after tone onset). When we analyzed this shorter interval, results for LP were similar to those for the longer, preregistered interval. For the preregistered prior, Bayesian one-sample *t* tests confirmed the presence of LP for response trials (BF_10_ > 45,000) and no-response trials (BF_10_ > 260,000). However, there was inconclusive evidence for or against a difference in mean LP amplitudes between response trials and no-response trials (BF_01_ = 2.5). In contrast, the BF for a default prior (Cauchy = 0.707) provided moderate evidence for no difference between response and no-response trials (BF_01_ = 6.5). Furthermore, as shown in Figure 3, the LP was more posterior than our preregistered electrodes. Therefore, we repeated the above analyses with a data-driven electrode selection (Wiens and Eklund, 2019). Results were similar to those with the preregistered electrode selection. For example, with the default prior, the evidence suggested no difference between response and no-response trials for LP for the preregistered interval (BF_01_ = 6.6) and the shorter interval (BF_01_ = 5.8).

As for AAN, we simulated the effect of larger sample sizes on the Bayes Factor with preregistered prior and electrodes. For the preregistered LP interval (350–550 ms), 130 or more subjects might yield moderate support for no difference between response and no-response trials (i.e., BF_01_ > 3 with 95% confidence). For the shorter LP interval (350–450 ms), 230 or more subjects might yield moderate support for no difference between response and no-response trials (i.e., BF_01_ > 3 with 95% confidence).

### Late Positivity: Source Localization

**Figure 5:**
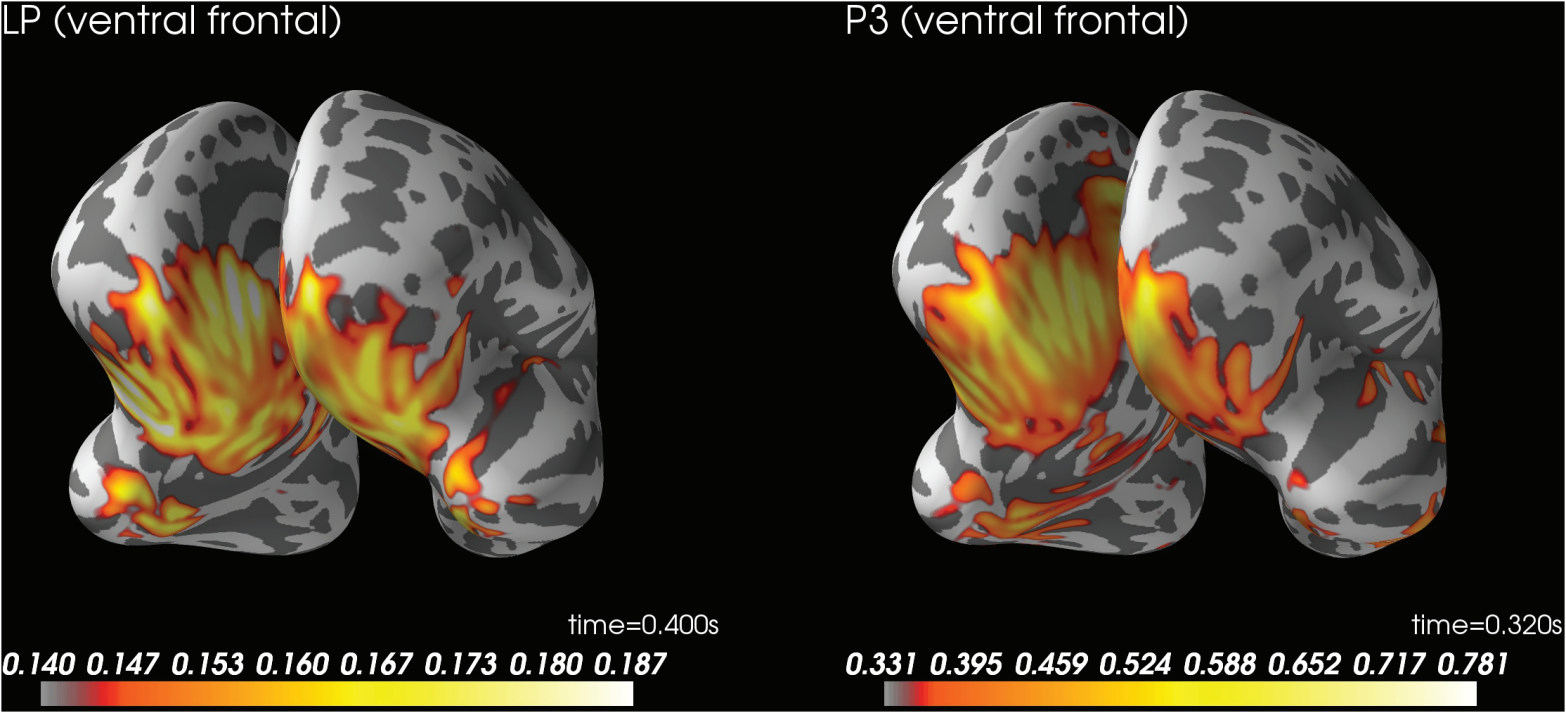
Source localization for LP at 400 ms after stimulus onset (left) and P3 to control trials rated as aware at 320 ms after stimulus onset (right). LP, late positivity.

Figure 5 shows source localization of LP. The source localization suggested activity in ventral temporal cortex and ventral prefrontal cortex. See supplementary files for videos of the time course (Wiens and Eklund, 2019).

## Discussion

The main results were that AAN and LP were present for response trials and for no-response trials, and that there was no apparent difference between response trials and no-response trials for either AAN or LP. Furthermore, the topography of AAN was characterized by a negativity over central electrodes, and source localization of this topography suggested sources in bilateral auditory cortices.

The present results replicate and extend our previous report of AAN (Eklund and Wiens, 2019). There was extreme evidence that AAN can be obtained whether or not subjects respond manually (Table 2). However, the preregistered Bayesian analyses provided inconclusive evidence regarding any effect of a response requirement on AAN (BF_01_ = 2.0). Whereas other studies have interpreted a nonsignificant difference as evidence for no effect, this reasoning is invalid (Amrhein et al., 2019). Therefore, we conducted Bayesian analyses to measure the evidence for or against an effect of response requirement. Despite a large sample size (*N* = 52), the evidential strength of our data was insufficient to distinguish between a hypothesis of no difference between response conditions (i.e., null hypothesis) and our preregistered hypothesis of a difference between −1 and +1 μV between conditions (i.e., alternative hypothesis or prior). From a Bayesian perspective, more data would be needed before either hypothesis could be supported. However, we did not continue data collection, for three reasons. First, we deemed it not worthwhile to further increase the sample size. When we used the data to simulate the effect of larger sample sizes, the simulation suggested that even 300 subjects would not be enough to obtain moderate evidence for no effect of a response requirement (BF_01_ = 2.7). Second, although our alternative hypothesis (of a difference between −1 and +1 μV between conditions) was reasonable, it was also arbitrary. When we considered the BF with a default prior that is recommended for standardized effects (Wagenmakers et al., 2017a,b), the evidential strength was moderate in support of no difference between conditions (BF_01_ = 5.9). Third, the mean difference in AAN between response conditions was only 0.17 μV, 95% CI [−0.53, 0.87]. This implies that the AAN difference is most likely close to zero but may differ by 0.87 μV between response conditions. Note that if this margin of error is considered relatively large, four times the number of subjects would be required to decrease its size by 50% (Wiens and Eklund, 2019).

In sum, a reasonable conclusion from the present findings is that AAN is unaffected by a response requirement. This finding matches previous reports of nonsignificant effects of a response requirement on VAN, which is the early neural correlate of awareness in vision (Koivisto et al., 2016; Ye and Lyu, 2019).

Because we used a high-density electrode array, we could characterize the topography of AAN as a central negativity. Source localization suggested sources in bilateral auditory cortices. These findings are consistent with a previous report that an awareness-related negativity was source localized to bilateral auditory cortices (Gutschalk et al., 2008). Taken together, these findings are consistent with recurrent processing theory (Lamme, 2006). As the brain receives sensory stimulation, automatic cascades of activation ensue called the feedforward sweep (Lamme and Roelfsema, 2000). The feedforward sweep is followed by localized recurrent loops of activations that occur within hierarchically early areas of the sensory cortices (localized recurrent processing). These recurrent loops then reach and include frontoparietal areas, resulting in global recurrent processing (Lamme, 2006). From this perspective, AAN and awareness-related negativity (Gutschalk et al., 2008) are neural correlates of localized recurrent processing in auditory cortices.

As for LP, the present results provide extreme evidence that LP can be obtained whether or not subjects respond manually (Table 2). Further, source localization of the LP suggested ventral temporal cortex and ventral prefrontal cortex. These results are consistent with the idea that LP reflects global recurrent processing (Lamme, 2006; Dehaene and Changeux, 2011). However, the preregistered Bayesian analyses provided only inconclusive evidence regarding a difference between response and no-response trials (BF_01_ = 2.5). When we used the data to simulate the effect of larger sample sizes, the simulation suggested that 120 subjects would be enough to obtain moderate evidence for no difference between response conditions (i.e., BF_01_ > 3). When we considered the BF with a default prior (Cauchy = 0.707), the evidential strength was moderate in support of no difference between conditions (BF_01_ > 5.5). Furthermore, the mean difference in LP between response conditions was only −0.16 μV, 95% CI [−0.68, 0.36]. In sum, a reasonable conclusion from the present findings is that LP is unaffected by a response requirement.

This conclusion does not match previous reports of significant effects of a response requirement on the LP in vision at an early interval between 350 and 450 ms (Koivisto et al., 2016) and (in an exploratory analysis) at a late interval between 450 and 650 ms (Ye and Lyu, 2019). Of course, it is possible that future research will show that the LP in hearing is affected by a response requirement. However, the present data provide an unbiased estimate of the effect because we preregistered both method and analyses to avoid the multiple comparison problem that is prone to yielding false positives in ERP research (Luck and Gaspelin, 2017).

The early neural correlates (AAN in hearing and VAN in vision) and the later neural correlates (LP in hearing and vision) may map onto different processes related to awareness. In terms of recurrent processing theory (Lamme, 2006), AAN and VAN may be indirect measures of local recurrent processing; thus, they index phenomenal consciousness, which refers to what it is like to have an experience (Block, 2005). Furthermore, LP may be an indirect measure of global recurrent processing; thus, it indexes access consciousness, which refers to reporting and introspecting about an experience (Block, 2005). However, in terms of global workspace theory (Dehaene and Changeux, 2011), AAN and VAN may capture only preconscious processing, whereas LP is an indirect measure of global recurrent processing; thus, it indexes both phenomenal and access consciousness (Cohen and Dennett, 2011; Naccache, 2018). The present findings do not resolve this discussion, but they show that both AAN and LP are neural correlates of auditory awareness that are not confounded by the requirement to respond manually when subjects report on their awareness.

## Data Availability

All datasets for this study are included in the manuscript/supplementary files.

## Ethics Statement

Ethical review and approval was not required for the study on human participants in accordance with the local legislation and institutional requirements. The patients/participants provided their written informed consent to participate in this study.

## Author Contributions

All authors designed the study and wrote the manuscript together. RE programmed the experiment and wrote the scripts to analyze the behavioral and EEG data. RE and BG collected the data.

### Conflict of Interest Statement

The authors declare that the research was conducted in the absence of any commercial or financial relationships that could be construed as a potential conflict of interest.

## Footnotes

^1^osf.io/zw8q7
